# Complex Generalized Synchronization and Parameter Identification of Nonidentical Nonlinear Complex Systems

**DOI:** 10.1371/journal.pone.0152099

**Published:** 2016-03-24

**Authors:** Shibing Wang, Xingyuan Wang, Bo Han

**Affiliations:** 1 Faculty of Electronic Information and Electrical Engineering, Dalian University of Technology, Dalian, 116024, China; 2 School of Computer and Information Engineering, Fuyang Normal University, Fuyang, 236041, China; Lanzhou university of Technology, CHINA

## Abstract

In this paper, generalized synchronization (GS) is extended from real space to complex space, resulting in a new synchronization scheme, complex generalized synchronization (CGS). Based on Lyapunov stability theory, an adaptive controller and parameter update laws are designed to realize CGS and parameter identification of two nonidentical chaotic (hyperchaotic) complex systems with respect to a given complex map vector. This scheme is applied to synchronize a memristor-based hyperchaotic complex Lü system and a memristor-based chaotic complex Lorenz system, a chaotic complex Chen system and a memristor-based chaotic complex Lorenz system, as well as a memristor-based hyperchaotic complex Lü system and a chaotic complex Lü system with fully unknown parameters. The corresponding numerical simulations illustrate the feasibility and effectiveness of the proposed scheme.

## Introduction

Since Fowler *et al*. proposed a complex Lorenz system in 1982 [1], modeling, analyses and synchronization of complex systems have attracted more and more attention in nonlinear science and technology fields, the reasons of which can be roughly summed up in the following two aspects. On the one hand, some physical systems and phenomena should be accurately modeled by complex systems, such as rotating fluids, detuned lasers, disk dynamos, electronic circuits, and so on [1–4]; on the other, due to the existence of complex variables which can double the number of variables, complex systems can generate complicated dynamical behaviors with strong unpredictability, and synchronization of complex systems has widely potential applications to many fields of physics, ecological systems, signal and information processing, and system identification, especially to secure communication for achieving higher transmission efficiency and anti-attack ability [5–7].

As we well know, chaos synchronization is the precondition of chaotic secure communication, digital cryptography, chaotic image encryption, *etc*. Since the pioneering work by Pecora and Carrol in 1990 [8], chaos synchronization of real systems has been extensively investigated theoretically and experimentally, while the synchronization of complex systems has been explored for less than a decade. In the beginning stages, some synchronization schemes were directly used to synchronize complex systems, such as complete synchronization (CS) [9–10], lag synchronization (LS) [7, 11], projective synchronization (PS) [12–13], phase synchronization (PhS) [14], combination synchronization [15], *etc*. Recently, some complex synchronization methods were presented based on their real versions. Liu *et al*. proposed a complex modified hybrid projective synchronization (CMHPS) scheme to synchronize complex Dadras systems, with different dimensions and complex parameters, up to a desired complex transformation matrix [16]. Wang *et al*. investigated a hybrid synchronization method containing complex modified projective synchronization and module-phase synchronization [17]. Sun *et al*. realized complex combination synchronization of three identical chaotic complex systems with complex scaling matrices [18]. Jiang *et al*. designed a general controller to achieve combination complex synchronization for fractional-order chaotic complex systems [19]. It is worth noting that Refs [20–21] have explored the synchronization issues of complex systems with unknown parameters which are likely to exist in practice. Zhang *et al*. investigated the complex modified projective synchronization (CMPS) and parameter identification of uncertain real chaotic systems and complex chaotic systems [20]. Liu *et al*. used an adaptive complex modified projective synchronization (ACMPS) method to synchronize two chaotic (hyperchaotic) complex systems up to a complex scaling matrix, and to estimate the unknown complex parameters successfully [21].

Based on the above-mentioned complex synchronization methods, the response complex systems can be synchronized with the drive complex systems up to the desired complex scaling matrices. Shall we further generalize these synchronization schemes and synchronize the complex systems with respect to a given complex functional relationship? That is, can generalized synchronization (GS) be extended to synchronize complex systems? Rulkov *et al*. firstly proposed the generalized synchronization, where two chaotic systems are said to be synchronized if a given functional relation can be realized between the variables of drive and response systems [22]. With different given functions, GS can degenerate to various PSs, antisynchronization (AS) and CS. Furthermore, the given functions are almost impossible to be predicted, which can enhance secure performance when GS is applied to chaotic secure communication. In the recent two decades, GS of chaotic or hyperchaotic real systems has been widely investigated. For instance, Refs [23–25] realized GS of different chaotic and hyperchaotic systems, while Refs [26–28] achieved adaptive generalized synchronization (AGS) and parameter identification of different chaotic systems with unknown parameters. However, to our best knowledge, up to now, there are few published achievements on CGS of nonidentical nonlinear complex systems. So, it is meaningful and challenging to extend GS from real systems to complex systems, and to realize CGS and parameter identification of chaotic and hyperchaotic complex systems with unknown parameters.

Motivated by the above discussions, this paper investigates CGS and parameter identification of different chaotic and hyperchaotic complex systems with unknown parameters. In practice, the parameters of some nonlinear systems cannot be exactly known, so we choose uncertain nonlinear complex systems as the research objects, and use adaptive control and Lyapunov stability theory to design CGS and parameter estimation scheme for them. In our proposed scheme, CGS is defined by extending GS from real space to complex space, and designed with consideration of error feedback control gains which are introduced to adjust converging velocity. Furthermore, according to the orders of the drive and response nonlinear complex systems (i.e., same-order, increased-order, and reduced-order), three different examples are presented to verify the correctness, feasibility, and efficiency of the proposed scheme.

The rest of this paper is organized as follows. The definition and design of CGS of nonidentical complex systems are given in Section 2. CGS and parameter identification of a memristor-based hyperchaotic complex Lü system and a memristor-based chaotic complex Lorenz system with the same orders, a chaotic complex Chen system and a memristor-based chaotic complex Lorenz system via increased order, as well as a memristor-based hyperchaotic complex Lü system and a chaotic complex Lü system via reduced order, are investigated theoretically and illustrated numerically in Section 3–5, respectively. Finally, some conclusions are drawn in Section 6.

## Design of CGS

### Definition of CGS

Consider the following nonidentical drive and response complex systems with fully unknown parameters

$$\dot{x}=F(x)\theta +f(x)$$ (1)

$$\dot{y}=G(y)\delta +g(y)+u(x,y)$$ (2)

where ***x*** = (*x*_1_, *x*_2_, ⋯*x*_*n*_)^*T*^ and ***y*** = (*y*_1_, *y*_2_, ⋯*x*_*m*_)^*T*^ are complex state vectors of the drive system (1) and response system (2) respectively, *x*_*k*_ = *x*_k,r_ + j*x*_k,i_(*k* = 1, ⋯, *n*), *y*_*k*_ = *y*_k,r_ + j*y*_k,i_(*k* = 1, ⋯, *m*), $\text{j}=\sqrt{-1}$, the subscripts r and i denote the real and image parts of the complex variables, vectors and matrices throughout this paper. ***θ***∈R^*p*^ and ***δ***∈R^*q*^ are real vectors of unknown parameters. ***F***(***x***)∈**C**^*n×p*^ and ***G***(***y***)∈**C**^*m×q*^ are complex matrices, ***F***(***x***) = ***F***_r_(***x***) + j***F***_i_(***x***), ***G***(***y***) = ***G***_r_(***y***) + j***G***_i_(***y***). ***f***(***x***)∈**C**^*n*^ and ***g***(***y***)∈**C**^*m*^ are vectors of nonlinear complex functions, and ***f***(***x***) = ***f***_r_(***x***) + j***f***_i_(***x***), ***g***(***y***) = ***g***_r_(***y***) + j***g***_i_(***y***). ***u***(***x*, *y***)∈**C**^*m*^ is the complex control vector, and ***u***(***x*, *y***) = ***u***_r_(***x*, *y***) + j***u***_i_(***x*, *y***).

**Remark 1** Some nonlinear complex systems can be formed as system (1), such as complex Lorenz system, complex Chen system, complex Lü system, memristor-based complex Lorenz system, memristor-based complex Lü system, and so on. For synchronizing such complex systems, the complex variables and functions could be divided into the real parts and imaginary parts.

**Definition 1** For the drive system (1) and response system (2), CGS is achieved if there exist a complex controller ***u***(***x*, *y***) and a given complex map ***ϕ***(***x***):**C**^*n*^→**C**^*m*^ such that

$$\underset{t\to \infty }{\text{lim}}\left\|y-\phi (x)\right\|=0$$ (3)

where ***ϕ***(***x***) = [*ϕ*_1_(***x***), *ϕ*_1_(***x***), ⋯*ϕ*_*m*_(***x***)]^T^ is a nonzero complex map vector whose elements are continuously differentiable complex functions of ***x***, and ***ϕ***(***x***) = ***ϕ***_r_(***x***) + j***ϕ***_i_(***x***).

**Remark 2** If ***ϕ***(***x***) = Θ***x***, some types of synchronization are special cases of CGS, such as complex modified hybrid projective synchronization (CMHPS) as Θ∈**C**^*m×n*^, complex modified projective synchronization (CMPS) as Θ = diag(*ϑ*_1_, *ϑ*_2_, ⋯*ϑ*_n_)∈**C**^*m×n*^, complex projective synchronization (CPS) as Θ = diag(*ϑ*, *ϑ*, ⋯*ϑ*)∈**C**^*n×n*^, modified hybrid projective synchronization (MHPS) as Θ∈**R**^*m×n*^, modified projective synchronization (MPS) as Θ = diag(*ϑ*_1_, *ϑ*_2_, ⋯*ϑ*_n_)∈**R**^*n×n*^, projective synchronization (PS) as Θ = diag(*ϑ*, *ϑ*, ⋯*ϑ*)∈**R**^*n×n*^, antisynchronization (AS) as Θ = diag(− 1, − 1, ⋯ − 1), and complete synchronization (CS) as Θ = diag(1, 1, ⋯1).

### General scheme of CGS and parameter identification

Define the complex CGS error vector as

$$\begin{array}{ll} e & =y-\phi (x)=e_{\text{r}}+\text{j}e_{\text{i}}\\ & =(y_{\text{r}}-\phi_{\text{r}}(x))+\text{j}(y_{\text{i}}-\phi_{\text{i}}(x)) \end{array}$$ (4)

where ***e*** = (*e*_1_, *e*_2_, ⋯*e*_*m*_)^*T*^∈**C**^*m*^, ***e***_r_ = (*e*_1,r_, *e*_2,r_, ⋯*e*_m,r_)^*T*^∈**R**^*m*^,***e***_i_ = (*e*_1,i_, *e*_2,i_, ⋯*e*_m,i_)^*T*^∈**R**^*m*^. By taking the derivative of Eq 4 with respect time, the CGS error dynamical system is obtained as

$$\dot{e}=\dot{y}-J(\phi )\dot{x}$$ (5)

where ***J***(*ϕ*)∈**C**^*m×n*^ is the Jacobian matrix of ***ϕ***(***x***), and ***J***(*ϕ*) = ***J***_r_(*ϕ*) + j***J***_i_(*ϕ*). By substituting Eqs 1 and 2 into Eq 5, Eq 5 can be represented by

$$\begin{array}{ll} \dot{e} & ={\dot{e}}_{\text{r}}+\text{j}{\dot{e}}_{\text{i}}\\ & =\left\{g_{\text{r}}(y)-J_{\text{r}}(\phi )f_{\text{r}}(x)+J_{\text{i}}(\phi )f_{\text{i}}(x)-[J_{\text{r}}(\phi )F_{\text{r}}(x)-J_{\text{i}}(\phi )F_{\text{i}}(x)]\theta +G_{\text{r}}(y)\delta +u_{\text{r}}(x,y)\right\}\\ & +\text{j}\left\{g_{\text{i}}(y)-J_{\text{r}}(\phi )f_{\text{i}}(x)-J_{\text{i}}(\phi )f_{\text{r}}(x)-[J_{\text{r}}(\phi )F_{\text{i}}(x)+J_{\text{i}}(\phi )F_{\text{r}}(x)]\theta +G_{\text{i}}(y)\delta +u_{\text{i}}(x,y)\right\} \end{array}$$ (6)

Therefore, the problem of CGS for two nonidentical complex systems (1) and (2) is transformed to the stability analysis of zero solution of the error dynamical system (6). Adaptive CGS scheme is given in Theorem 1 and is proved based on Lyapunov stability theory.

**Theorem 1** For a given complex map vector ***ϕ***(***x***), CGS and parameter identification of the response system (2) and the drive system (1) can be achieved if the complex adaptive controller and the parameter update laws are designed as

$$\begin{array}{ll} u(x,y) & =u_{\text{r}}(x,y)+\text{j}u_{\text{i}}(x,y)\\ & =\left\{-g_{\text{r}}(y)+J_{\text{r}}(\phi )f_{\text{r}}(x)-J_{\text{i}}(\phi )f_{\text{i}}(x)+[J_{\text{r}}(\phi )F_{\text{r}}(x)-J_{\text{i}}(\phi )F_{\text{i}}(x)]\hat{\theta }-G_{\text{r}}(y)\hat{\delta }-Ke_{\text{r}}\right\}\\ & +\text{j}\left\{-g_{\text{i}}(y)+J_{\text{r}}(\phi )f_{\text{i}}(x)+J_{\text{i}}(\phi )f_{\text{r}}(x)+[J_{\text{r}}(\phi )F_{\text{i}}(x)+J_{\text{i}}(\phi )F_{\text{r}}(x)]\hat{\theta }-G_{\text{i}}(y)\hat{\delta }-Ke_{\text{i}}\right\} \end{array}$$ (7)

$$\{\begin{array}{ll} \dot{\tilde{\theta }}=\dot{\theta } & =-{\left[J_{\text{r}}(\phi )F_{\text{r}}(x)-J_{\text{i}}(\phi )F_{\text{i}}(x)\right]}^{T}e_{\text{r}}-{\left[J_{\text{r}}(\phi )F_{\text{i}}(x)+J_{\text{i}}(\phi )F_{\text{r}}(x)\right]}^{T}e_{\text{i}}-K_{\theta }\tilde{\theta }\\ \dot{\tilde{\delta }}=\dot{\hat{\delta }} & ={\left[G_{\text{r}}(y)\right]}^{T}e_{\text{r}}+{\left[G_{\text{i}}(y)\right]}^{T}e_{\text{i}}-K_{\delta }\tilde{\delta } \end{array}$$ (8)

where $\hat{\theta }$ and $\hat{\delta }$ denote the estimated parameter vectors of ***θ*** and ***δ***, $\tilde{\theta }=\hat{\theta }-\theta$ and $\tilde{\delta }=\hat{\delta }-\delta$ denote the parameter error vectors. ***K*** = diag(*k*_1_, *k*_2_⋯*k*_*m*_), ***K***_*θ*_ = diag(*k*_θ, 1_, *k*_θ, 2_⋯*k*_θ, p_) and **K**_*δ*_ = diag(*k*_δ, 1_, *k*_δ, 2_⋯*k*_*δ*, *q*_) are error feedback control strength whose elements are all positive constants, which can adjust converging velocity.

**Proof** We introduce a positive Lyapunov function as

$$V(t)=\frac{1}{2}[{\left(e_{\text{r}}\right)}^{T}e_{\text{r}}+{\left(e_{\text{i}}\right)}^{T}e_{\text{i}}+{\tilde{\theta }}^{T}\tilde{\theta }+{\tilde{\delta }}^{T}\tilde{\delta }]$$ (9)

The time derivative of ***V***(*t*) along the trajectories of the error dynamical system (6) is calculated as

$$\begin{array}{ll} \dot{V}(t) & ={\left({\dot{e}}_{\text{r}}\right)}^{T}e_{\text{r}}+{\left({\dot{e}}_{\text{i}}\right)}^{T}e_{\text{i}}+{\tilde{\theta }}^{T}\dot{\tilde{\theta }}+{\tilde{\delta }}^{T}\dot{\tilde{\delta }}\\ & ={\left\{g_{\text{r}}(y)-J_{\text{r}}(\phi )f_{\text{r}}(x)+J_{\text{i}}(\phi )f_{\text{i}}(x)-[J_{\text{r}}(\phi )F_{\text{r}}(x)-J_{\text{i}}(\phi )F_{\text{i}}(x)]\theta +G_{\text{r}}(y)\delta +u_{\text{r}}(x,y)\right\}}^{T}e_{\text{r}}\\ & +{\left\{g_{\text{i}}(y)-J_{\text{r}}(\phi )f_{\text{i}}(x)-J_{\text{i}}(\phi )f_{\text{r}}(x)-[J_{\text{r}}(\phi )F_{\text{i}}(x)+J_{\text{i}}(\phi )F_{\text{r}}(x)]\theta +G_{\text{i}}(y)\delta +u_{\text{i}}(x,y)\right\}}^{T}e_{\text{i}}\\ & +{\tilde{\theta }}^{T}\dot{\tilde{\theta }}+{\tilde{\delta }}^{T}\dot{\tilde{\delta }} \end{array}$$ (10)

Substituting Eqs 7 and 8 into Eq 10, then

$$\begin{array}{ll} \dot{V}(t) & ={\left\{[J_{\text{r}}(\phi )F_{\text{r}}(x)-J_{\text{i}}(\phi )F_{\text{i}}(x)]\tilde{\theta }-G_{\text{r}}(y)\tilde{\delta }-Ke_{\text{r}}\right\}}^{T}e_{\text{r}}\\ & +{\left\{[J_{\text{r}}(\phi )F_{\text{i}}(x)+J_{\text{i}}(\phi )F_{\text{r}}(x)]\tilde{\theta }-G_{\text{i}}(y)\tilde{\delta }-Ke_{\text{i}}\right\}}^{T}e_{\text{i}}\\ & -{\tilde{\theta }}^{T}\{{\left[J_{\text{r}}(\phi )F_{\text{r}}(x)-J_{\text{i}}(\phi )F_{\text{i}}(x)\right]}^{T}e_{\text{r}}+{\left[J_{\text{r}}(\phi )F_{\text{i}}(x)+J_{\text{i}}(\phi )F_{\text{r}}(x)\right]}^{T}e_{\text{i}}-K_{\theta }\tilde{\theta }\}\;\\ & +{\tilde{\delta }}^{T}\{{\left[G_{\text{r}}(y)\right]}^{T}e_{\text{r}}+{\left[G_{\text{i}}(y)\right]}^{T}e_{\text{i}}-K_{\delta }\tilde{\delta }\}\\ & =-{\left(e_{\text{r}}\right)}^{T}Ke_{\text{r}}-{\left(e_{\text{i}}\right)}^{T}Ke_{\text{i}}-{\tilde{\theta }}^{T}K_{\theta }\tilde{\theta }-{\tilde{\delta }}^{T}K_{\delta }\tilde{\delta }\\ & <0 \end{array}$$ (11)

Based on Lyapunov stability theory, since ***V***(*t*) and $\dot{V}(t)$ are positive and negative respectively, the CGS errors and the parameter errors asymptotically converge to zero as the time tends to infinity, i.e., $\underset{t\to \infty }{\text{lim}}e_{\text{r}}(t)=0$,$\underset{t\to \infty }{\text{lim}}e_{\text{i}}(t)=0$, $\underset{t\to \infty }{\text{lim}}\tilde{\theta }(t)=0$ and $\underset{t\to \infty }{\text{lim}}\tilde{\delta }(t)=0$, which indicate that CGS and parameter identification are realized. The proof is completed.

## CGS of a Memristor-Based Hyperchaotic Complex Lü System and a Memristor-Based Chaotic Complex Lorenz System (*n* = *m*)

**Fig 1:**
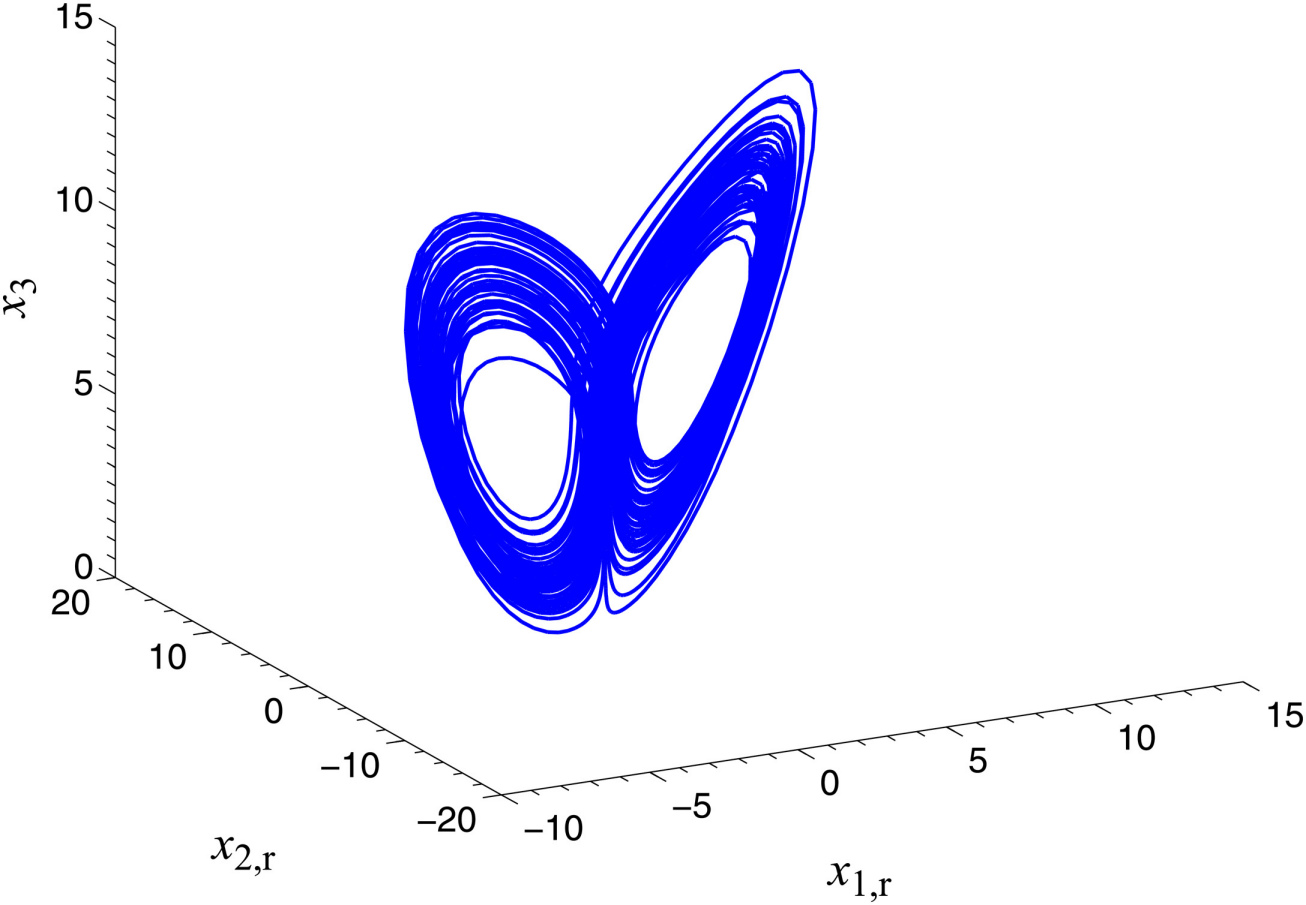
Hyperchaotic attractor of the memristor-based complex Lü system.

In this section, we investigate CGS of two nonidentical complex systems with the same orders. On the basis of a memristor-based hyperchaotic Lü system proposed in [29], a memristor-based hyperchaotic complex Lü system is introduced as the drive system, which is described as

$$\left\{ \begin{array}{l} {\dot{x}}_{1}=a_{1}(x_{2}-x_{1})\\ {\dot{x}}_{2}=-x_{1}x_{3}+a_{2}x_{2}-a_{3}(\alpha_{1}+3\beta_{1}x_{4}^{2})x_{1}\\ {\dot{x}}_{3}=\frac{1}{2}({\bar{x}}_{1}x_{2}+x_{1}{\bar{x}}_{2})-a_{4}x_{3}\\ {\dot{x}}_{4}=\frac{1}{2}({\bar{x}}_{1}+x_{1}) \end{array}\right.$$ (12)

where *x*_1_, *x*_2_∈C, *x*_3_, *x*_4_∈R, ${\bar{x}}_{1}\;,\;{\bar{x}}_{2}\;\in \text{C}$ denote the complex conjugate variables of *x*_1_, *x*_2_. *a*_1_, *a*_2_, *a*_3_ and *a*_4_ are unknown real parameters, *α*_1_ and *β*_1_ are considered as known positive constants. When *α*_1_ = 4, *β*_1_ = 0.01, *a*_1_ = 36, *a*_2_ = 20, *a*_3_ = 3.2, *a*_4_ = 3 and *x*(0) = [−1 + 2j, 1 + j, 2, −1]^*T*^, the Lyapunov exponents of system (12) are calculated as (0.262, 0.103, 0, 0, -15.764, -17.553), and a hyperchaotic attractor is plotted in Fig 1.

**Fig 2:**
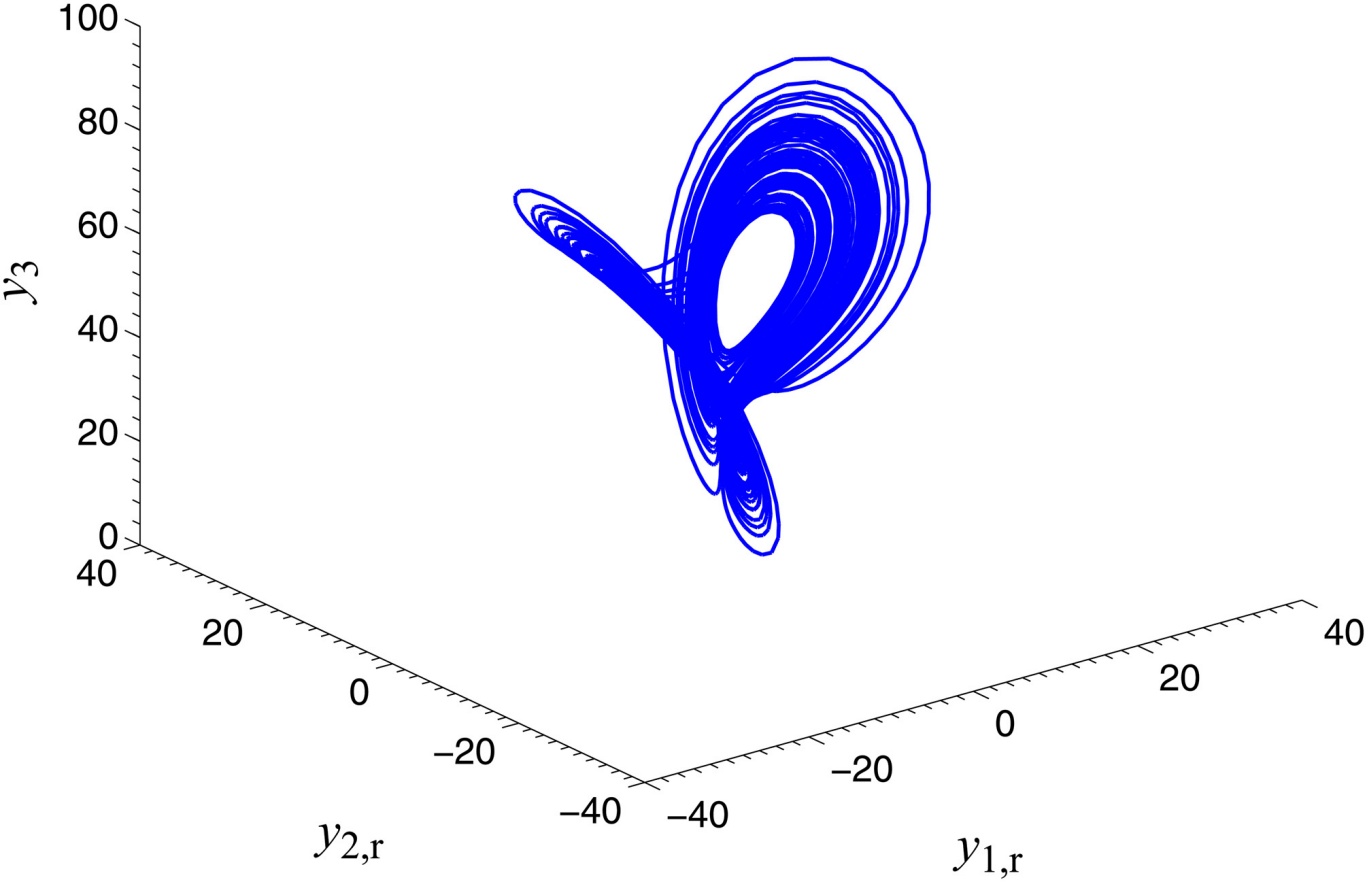
Chaotic attractor of the memristor-based complex Lorenz system.

A memristor-based chaotic complex Lorenz system, proposed in [30], is introduced as the response system

$$\{\begin{array}{l} {\dot{y}}_{1}=-b_{1}y_{1}-(\alpha_{2}+3\beta_{2}y_{4}^{2})y_{1}+b_{2}y_{2}+u_{1}\\ {\dot{y}}_{2}=b_{3}y_{1}-y_{2}-y_{1}y_{3}+u_{2}\\ {\dot{y}}_{3}=\frac{1}{2}({\bar{y}}_{1}y_{2}+y_{1}{\bar{y}}_{2})-b_{4}y_{3}+u_{3}\\ {\dot{y}}_{4}=-\frac{1}{2}({\bar{y}}_{1}+y_{1})+u_{4} \end{array}$$ (13)

where *y*_1_, *y*_2_∈C, *y*_3_, *y*_4_∈R, *b*_1_, *b*_2_, *b*_3_ and *b*_4_ are unknown real parameters, *α*_2_ and *β*_2_ are considered as known positive constants. *u*_1_, *u*_2_, u_3_ and *u*_4_ are controllers. When *α*_2_ = 0.67 × 10^−3^, *β*_2_ = 0.02 × 10^−3^, *b*_1_ = 8, *b*_2_ = 11, *b*_3_ = 50, *b*_4_ = 8/3 and *y*(0) = [2, 1 + 4j, 0.1, 0]^*T*^, the system (13) operates in chaotic orbits without control, as shown in Fig 2.

The drive system (12) and response system (13) can be rewritten with the form of Eqs 1 and 2, where ***θ*** = [*a*_1_, *a*_2_, *a*_3_, *a*_4_]^*T*^, ***δ*** = [*b*_1_, *b*_2_, *b*_3_, *b*_4_]^*T*^, ***u*** = [*u*_1_, *u*_2_, *u*_3_, *u*_4_]^*T*^, and

$$F(x)=\left[\begin{array}{cccc} x_{\text{2}}-x_{\text{1}} & 0 & 0 & 0\\ 0 & x_{\text{2}} & -(\alpha_{1}+3\beta_{1}x_{4}^{2})x_{\text{1}} & 0\\ 0 & 0 & 0 & -x_{3}\\ 0 & 0 & 0 & 0 \end{array}\right],\;f(x)=\left[\begin{array}{c} 0\\ -x_{\text{1}}x_{3}\\ (x_{\text{1}}{\bar{x}}_{\text{2}}+{\bar{x}}_{\text{1}}x_{\text{2}})/2\\ (x_{\text{1}}+{\bar{x}}_{\text{1}})/2 \end{array}\right]$$

$$G(y)=\left[\begin{array}{cccc} -y_{\text{1}} & y_{\text{2}} & 0 & 0\\ 0 & 0 & y_{\text{1}} & 0\\ 0 & 0 & 0 & -y_{3}\\ 0 & 0 & 0 & 0 \end{array}\right],\;g(y)=\left[\begin{array}{c} -(\alpha_{2}+3\beta_{2}y_{4}^{2})y_{1}\\ -y_{\text{2}}-y_{\text{1}}y_{3}\\ (y_{\text{1}}{\bar{y}}_{\text{2}}+{\bar{y}}_{\text{1}}y_{\text{2}})/2\\ -(y_{\text{1}}+{\bar{y}}_{\text{1}})/2 \end{array}\right]$$

The complex map vector is given by

$$\phi (x)={\left[x_{1}+\text{j}x_{2},(2-\text{j})x_{2},x_{3}+x_{4},x_{4}^{2}\right]}^{T}$$ (14)

The Jacobian matrix of the complex map vector is calculated as

$$J(\phi )=\left[\begin{array}{cccc} 1 & \text{j} & 0 & 0\\ 0 & 2-\text{j} & 0 & 0\\ 0 & 0 & 1 & 1\\ 0 & 0 & 0 & 2x_{4} \end{array}\right]$$ (15)

According to Eqs 7 and 8, the complex adaptive controller and parameter estimator can be obtained as

$$\begin{array}{c} u_{1}=[(\alpha_{2}+3\beta_{2}y_{4}^{2})y_{\text{1,r}}+x_{\text{1,i}}x_{3}+(x_{\text{2,r}}-x_{\text{1,r}}){\hat{a}}_{1}-x_{\text{2,i}}{\hat{a}}_{2}+(\alpha_{1}+3\beta_{1}x_{4}^{2})x_{\text{1,i}}{\hat{a}}_{3}+y_{\text{1,r}}{\hat{b}}_{1}-y_{\text{2,r}}{\hat{b}}_{2}-k_{1}e_{\text{1,r}}]\\ \;+\text{j }[(\alpha_{2}+3\beta_{2}y_{4}^{2})y_{\text{1,i}}-x_{\text{1,r}}x_{3}+(x_{\text{2,i}}-x_{\text{1,i}}){\hat{a}}_{1}+x_{\text{2,r}}{\hat{a}}_{2}-(\alpha_{1}+3\beta_{1}x_{4}^{2})x_{\text{1,r}}{\hat{a}}_{3}+y_{\text{1,i}}{\hat{b}}_{1}-y_{\text{2,i}}{\hat{b}}_{2}-k_{1}e_{\text{1,i}}]\\ u_{2}=[y_{\text{2,r}}+y_{\text{1,r}}y_{3}-(2x_{\text{1,r}}+x_{\text{1,i}})x_{3}+(2x_{\text{2,r}}+x_{\text{2,i}}){\hat{a}}_{2}-(2x_{\text{1,r}}+x_{\text{1,i}})(\alpha_{1}+3\beta_{1}x_{4}^{2}){\hat{a}}_{3}-y_{\text{1,r}}{\hat{b}}_{3}-k_{2}e_{\text{2,r}}]\\ \;+\text{j }[y_{\text{2,i}}+y_{\text{1,i}}y_{3}-(2x_{\text{1,i}}-x_{\text{1,r}})x_{3}+(2x_{\text{2,i}}-x_{\text{2,r}}){\hat{a}}_{2}-(2x_{\text{1,i}}-x_{\text{1,r}})(\alpha_{1}+3\beta_{1}x_{4}^{2}){\hat{a}}_{3}-y_{\text{1,i}}{\hat{b}}_{3}-k_{2}e_{\text{2,i}}]\\ u_{3}=-y_{\text{1,r}}y_{\text{2,r}}-y_{\text{1,i}}y_{\text{2,i}}+x_{\text{1,r}}x_{\text{2,r}}+x_{\text{1,i}}x_{\text{2,i}}+x_{\text{1,r}}-x_{3}{\hat{a}}_{4}+y_{\text{3}}{\hat{b}}_{4}-k_{3}e_{\text{3}}\\ u_{4}=y_{\text{1,r}}+2x_{\text{1,r}}x_{\text{4}}-k_{4}e_{\text{4}} \end{array}$$ (16)

$$\begin{array}{l} {\dot{\hat{a}}}_{1}=-(x_{\text{2,r}}-x_{\text{1,r}})e_{\text{1,r}}-(x_{\text{2,i}}-x_{\text{1,i}})e_{\text{1,i}}-k_{\theta ,1}({\hat{a}}_{1}-a_{1})\\ {\dot{\hat{a}}}_{2}=x_{\text{2,i}}e_{\text{1,r}}-x_{\text{2,r}}e_{\text{1,i}}-(2x_{\text{2,r}}+x_{\text{2,i}})e_{\text{2,r}}-(2x_{\text{2,i}}-x_{\text{2,r}})e_{\text{2,i}}-k_{\theta ,2}({\hat{a}}_{2}-a_{2})\\ {\dot{\hat{a}}}_{3}=[-x_{\text{1,i}}e_{\text{1,r}}+x_{\text{1,r}}e_{\text{1,i}}+(2x_{\text{1,r}}+x_{\text{1,i}})e_{\text{2,r}}+(2x_{\text{1,i}}-x_{\text{1,r}})e_{\text{2,i}}](\alpha_{1}+3\beta_{1}x_{4}^{2})-k_{\theta ,3}({\hat{a}}_{3}-a_{3})\\ {\dot{\hat{a}}}_{4}=x_{\text{3}}e_{\text{3}}-k_{\theta ,4}({\hat{a}}_{4}-a_{4}) \end{array}$$ (17)

$$\begin{array}{l} {\dot{\hat{b}}}_{1}=-y_{\text{1,r}}e_{\text{1,r}}-y_{\text{1,i}}e_{\text{1,i}}-k_{\delta ,1}({\hat{b}}_{1}-b_{1})\\ {\dot{\hat{b}}}_{2}=y_{\text{2,r}}e_{\text{1,r}}+y_{\text{2,i}}e_{\text{1,i}}-k_{\delta ,2}({\hat{b}}_{2}-b_{2})\\ {\dot{\hat{b}}}_{3}=y_{\text{1,r}}e_{\text{2,r}}+y_{\text{1,i}}e_{\text{2,i}}-k_{\delta ,3}({\hat{b}}_{3}-b_{3})\\ {\dot{\hat{b}}}_{4}=-y_{\text{3}}e_{\text{3}}-k_{\delta ,4}({\hat{b}}_{4}-b_{4}) \end{array}$$ (18)

where *e*_1,r_ = *y*_1,r_ − *x*_1,r_+*x*_2,i_, *e*_1,i_ = *y*_1,i_ − *x*_1,i_ − *x*_2,r_, *e*_2,r_ = *y*_2,r_ − 2*x*_2,r_ − *x*_2,i_, *e*_2,i_ = *y*_2,i_ − 2*x*_2,i_+*x*_2,r_, *e*_3_ = *y*_3_ − *x*_3_ − *x*_4_, $e_{\text{4}}=y_{\text{4}}-x_{{}_{\text{4}}}^{2}$.

**Fig 3:**
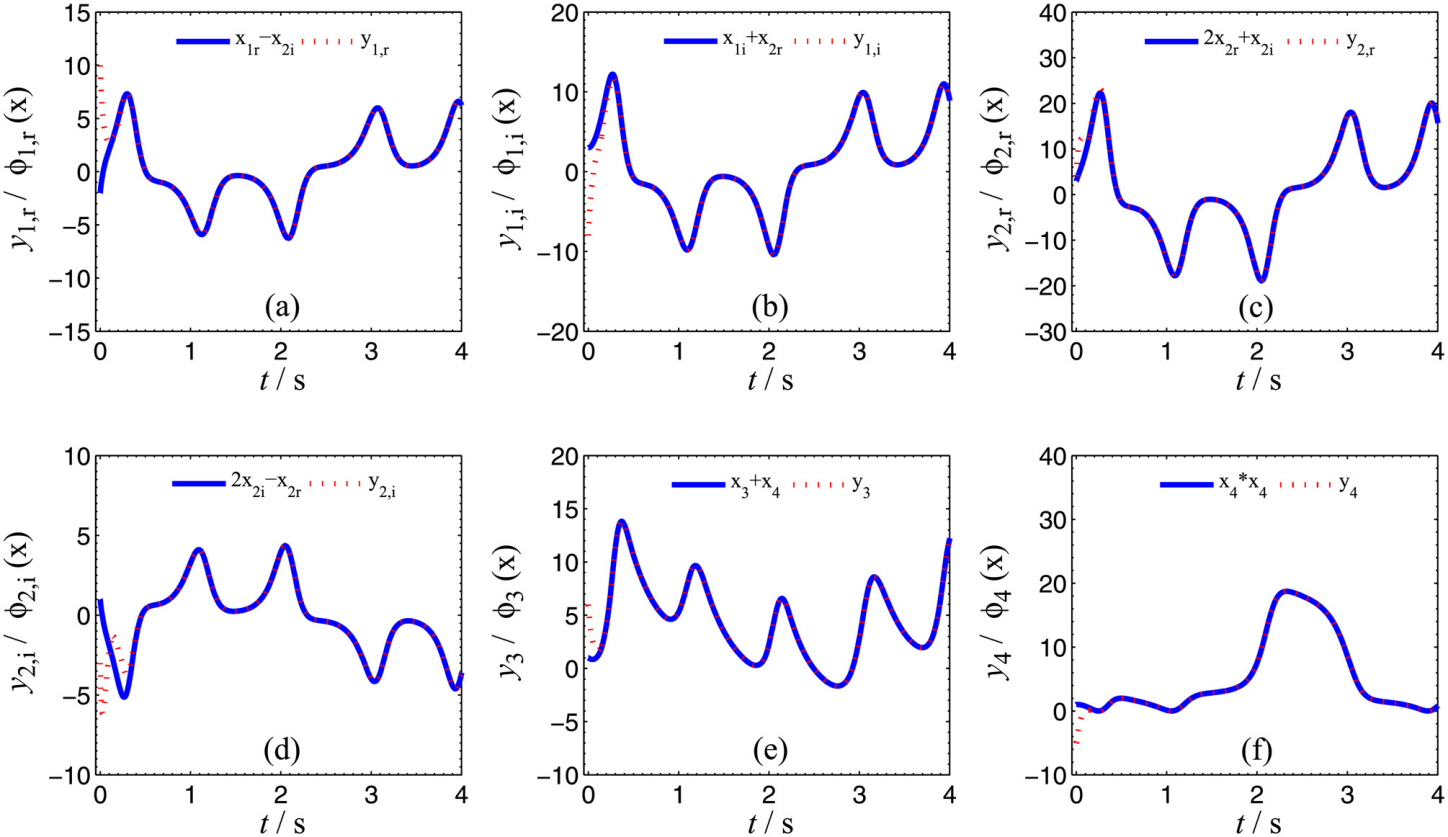
CGS process of systems (12) and (13) with respect to the given complex map vector. **$\phi (x)={\left[x_{1}+\text{j }x_{2},\;(2-\text{j })x_{2},\;x_{3}+x_{4},\;x_{4}^{2}\right]}^{T}$**. (a) *x*_1,r_ − *x*_2,i_, *y*_1,r_; (b) *x*_1r_ − *x*_2,r_, *y*_1,i_; (c) 2*x*_2,r_+*x*_2,i_, *y*_2,r_; (d) 2*x*_2,i_ − *x*_2,r_, *y*_2,i_; (e) *x*_3_+*x*_4_, *y*_3_; (f)$x_{{}_{4}}^{2},\;y_{\text{4}}$

**Fig 4:**
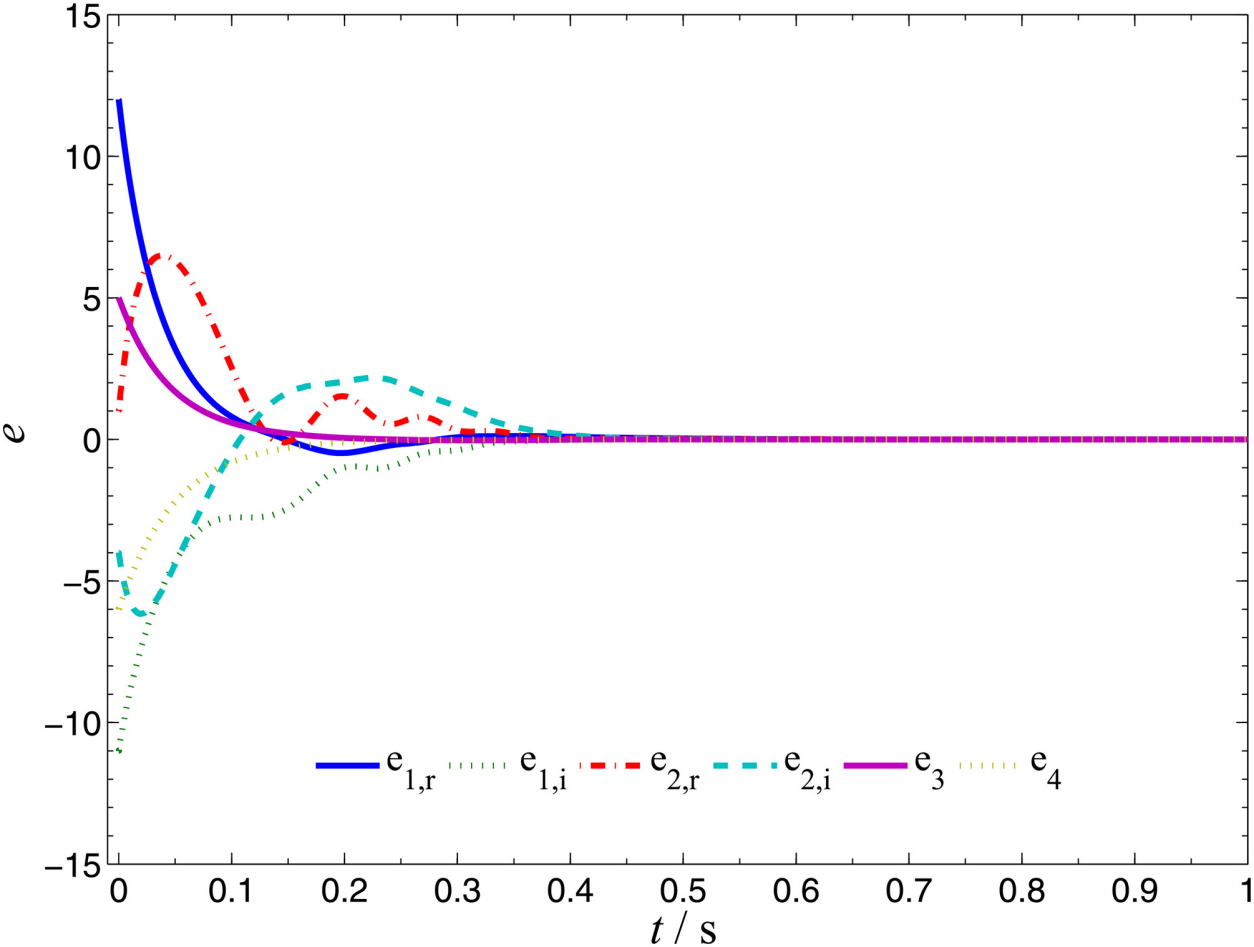
CGS errors of systems (12) and (13).

**Fig 5:**
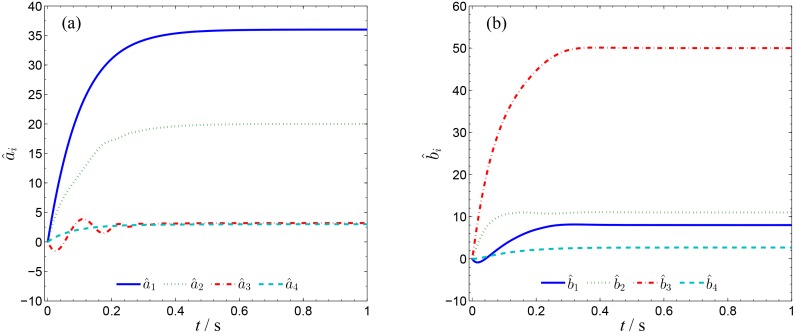
Identification of unknown parameters of systems (12) and (13). (a) ${\hat{a}}_{\text{1}},\;{\hat{a}}_{\text{2}},\;{\hat{a}}_{\text{3}},\;{\hat{a}}_{\text{4}}\;$; (b) ${\hat{b}}_{\text{1}},\;{\hat{b}}_{\text{2}},\;{\hat{b}}_{\text{3}},\;{\hat{b}}_{\text{4}}\;$.

In order to verify the validity and effectiveness of CGS between system (12) and (13) with respect to the complex vector (14), ODE45 algorithm is used to solve the systems based on Matlab 2013a. The values of known parameters are *α*_1_ = 4, *β*_1_ = 0.01, *α*_2_ = 0.67 × 10^−3^, *β*_2_ = 0.02 × 10^−3^, the true values of unknown parameters are *θ* = [36, 20, 3.2, 3]^*T*^, *δ* = [8, 11, 50, 8/3]^*T*^. The initial conditions of system (12) and (13) are randomly selected as ***x***(0) = [−1 + 2j, 1 + j, 2, −1]^*T*^, ***y***(0) = [10 − 8j, 4 − 3j, 6, −5]^*T*^. The initial values of all unknown parameters are randomly chosen as zero, and the control strength is set as ***K*** = diag(20, 20, 20, 20), ***K***_*θ*_ = diag(10, 10, 10, 10), ***K***_*δ*_ = diag(10, 10, 10, 10). The corresponding simulation results are shown in Figs 3, 4 and 5. The CGS process is plotted in Fig 3, which indicate that the response system (13) is synchronized with the drive system (12) with respect to the given complex map vector (14). The synchronization errors, as shown in Fig 4, converge to zero in a short time. Fig 5 shows the identifying processes of unknown parameters, which indicates that the estimated values tend to be their true values adaptively, i.e., $\hat{\theta }\to {\left[36,\;20,\;3.2,\;3\right]}^{T}$ and $\hat{\delta }\to {\left[8,\;11,\;50,\;2.667\right]}^{T}$.

## CGS of a Chaotic Complex Chen System and a Memristor-Based Chaotic Complex Lorenz System (*n*<*m*)

**Fig 6:**
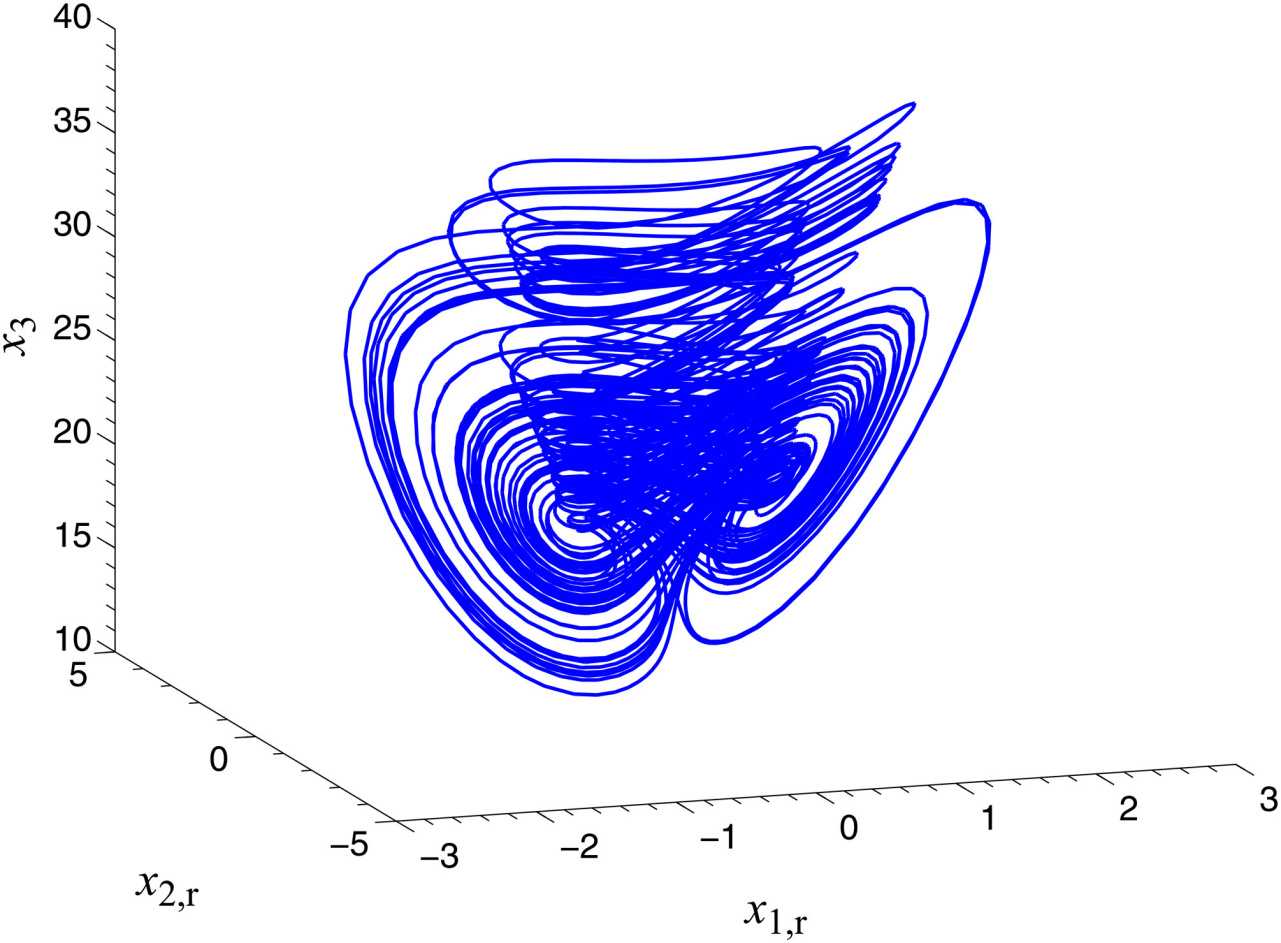
Chaotic attractor of the complex Chen system.

In this section, we investigate CGS of two nonidentical complex systems via increased order. A chaotic complex Chen system, investigated in [9], is introduced as the complex drive system, which is described as

$$\{\begin{array}{l} {\dot{x}}_{1}=c_{1}(x_{2}-x_{1})\\ {\dot{x}}_{2}=(c_{2}-c_{1})x_{1}-x_{1}x_{3}+c_{2}x_{2}\\ {\dot{x}}_{3}=\frac{1}{2}({\bar{x}}_{1}x_{2}+x_{1}{\bar{x}}_{2})-c_{3}x_{3} \end{array}$$ (19)

where *x*_1_, *x*_2_∈C, *x*_3_∈R, *c*_1_, *c*_2_ and *c*_3_ are unknown real parameters. When *c*_1_ = 27, *c*_2_ = 23, *c*_3_ = 1, and *x*(0) = [−3 − 2j, −1 − 5j, −4]^*T*^, the complex Chen system (19) operates in chaotic orbits, as shown in Fig 6. The memristor-based chaotic complex Lorenz system, i.e., system (13), is also served as the complex response system.

The drive system (19) and response system (13) can be rewritten with the form of Eqs 1 and 2, where ***θ*** = [*c*_1_, *c*_2_, *c*_3_]^*T*^, ***δ*** = [*b*_1_, *b*_2_, *b*_3_, *b*_4_]^*T*^, ***u*** = [*u*_1_, *u*_2_, *u*_3_, *u*_4_]^*T*^, and

$$F(x)=\left[\begin{array}{ccc} x_{\text{2}}-x_{\text{1}} & 0 & 0\\ -x_{\text{1}} & x_{\text{2}}-x_{\text{1}} & 0\\ 0 & 0 & -x_{3} \end{array}\right],\;f(x)=\left[\begin{array}{c} 0\\ -x_{\text{1}}x_{3}\\ (x_{\text{1}}{\bar{x}}_{\text{2}}+{\bar{x}}_{\text{1}}x_{\text{2}})/2 \end{array}\right]$$

$$G(y)=\left[\begin{array}{cccc} -y_{\text{1}} & y_{\text{2}} & 0 & 0\\ 0 & 0 & y_{\text{1}} & 0\\ 0 & 0 & 0 & -y_{3}\\ 0 & 0 & 0 & 0 \end{array}\right],\;g(y)=\left[\begin{array}{c} -(\alpha_{2}+3\beta_{2}y_{4}^{2})y_{1}\\ -y_{\text{2}}-y_{\text{1}}y_{3}\\ (y_{\text{1}}{\bar{y}}_{\text{2}}+{\bar{y}}_{\text{1}}y_{\text{2}})/2\\ -(y_{\text{1}}+{\bar{y}}_{\text{1}})/2 \end{array}\right]$$

The complex map vector is given by

$$\phi (x)={\left[-\text{j}x_{1},-\text{j}x_{2},-x_{3},x_{3}\right]}^{T}$$ (20)

The Jacobian matrix of the complex map vector is calculated as

$$J(\phi )=\left[\begin{array}{lll} -\text{j} & 0 & 0\\ 0 & -\text{j} & 0\\ 0 & 0 & -1\\ 0 & 0 & 1 \end{array}\right]$$ (21)

According to Eqs 7 and 8, the complex adaptive controller and parameter estimator can be obtained as

$$\begin{array}{c} u_{1}=[(\alpha_{2}+3\beta_{2}y_{4}^{2})y_{\text{1,r}}+(x_{\text{2,i}}-x_{\text{1,i}}){\hat{c}}_{1}+y_{\text{1,r}}{\hat{b}}_{1}-y_{\text{2,r}}{\hat{b}}_{2}-k_{1}e_{\text{1,r}}]\\ \;+\text{j }[(\alpha_{2}+3\beta_{2}y_{4}^{2})y_{\text{1,i}}-(x_{\text{2,r}}-x_{\text{1,r}}){\hat{c}}_{1}+y_{\text{1,i}}{\hat{b}}_{1}-y_{\text{2,i}}{\hat{b}}_{2}-k_{1}e_{\text{1,i}}]\\ u_{2}=[y_{\text{2,r}}+y_{\text{1,r}}y_{3}-x_{\text{1,i}}x_{3}-x_{\text{1,i}}{\hat{c}}_{1}+(x_{\text{2,i}}+x_{\text{1,i}}){\hat{c}}_{2}-y_{\text{1,r}}{\hat{b}}_{3}-k_{2}e_{\text{2,r}}]\\ \;+\text{j }[y_{\text{2,i}}+y_{\text{1,i}}y_{3}+x_{\text{1,r}}x_{3}+x_{\text{1,r}}{\hat{c}}_{1}-(x_{\text{2,r}}+x_{\text{1,r}}){\hat{c}}_{2}-y_{\text{1,i}}{\hat{b}}_{3}-k_{2}e_{\text{2,i}}]\\ u_{3}=-y_{\text{1,r}}y_{\text{2,r}}-y_{\text{1,i}}y_{\text{2,i}}-x_{\text{1,r}}x_{\text{2,r}}-x_{\text{1,i}}x_{\text{2,i}}+x_{3}{\hat{c}}_{3}+y_{\text{3}}{\hat{b}}_{4}-k_{3}e_{\text{3}}\\ u_{4}=y_{\text{1,r}}+x_{\text{1,r}}x_{\text{2,r}}+x_{\text{1,i}}x_{\text{2,i}}-x_{3}{\hat{c}}_{3}-k_{4}e_{\text{4}} \end{array}$$ (22)

$$\begin{array}{l} {\dot{\hat{c}}}_{1}=-(x_{\text{2,i}}-x_{\text{1,i}})e_{\text{1,r}}+(x_{\text{2,r}}-x_{\text{1,r}})e_{\text{1,i}}+x_{\text{1,i}}e_{\text{2,r}}-x_{\text{1,r}}e_{\text{2,i}}-k_{\theta 1}({\hat{c}}_{1}-c_{1})\\ {\dot{\hat{c}}}_{2}=-(x_{\text{2,i}}+x_{\text{1,i}})e_{\text{2,r}}+(x_{\text{2,r}}+x_{\text{1,r}})e_{\text{2,i}}-k_{\theta 2}({\hat{c}}_{2}-c_{2})\\ {\dot{\hat{c}}}_{3}=-x_{\text{3}}e_{\text{3}}+x_{\text{3}}e_{\text{4}}-k_{\theta 3}({\hat{c}}_{3}-c_{3}) \end{array}$$ (23)

$$\begin{array}{l} {\dot{\hat{b}}}_{1}=-y_{\text{1,r}}e_{\text{1,r}}-y_{\text{1,i}}e_{\text{1,i}}-k_{\delta 1}({\hat{b}}_{1}-b_{1})\\ {\dot{\hat{b}}}_{2}=y_{\text{2,r}}e_{\text{1,r}}+y_{\text{2,i}}e_{\text{1,i}}-k_{\delta 2}({\hat{b}}_{2}-b_{2})\\ {\dot{\hat{b}}}_{3}=y_{\text{1,r}}e_{\text{2,r}}+y_{\text{1,i}}e_{\text{2,i}}-k_{\delta 3}({\hat{b}}_{3}-b_{3})\\ {\dot{\hat{b}}}_{4}=-y_{\text{3}}e_{\text{3}}-k_{\delta 4}({\hat{b}}_{4}-b_{4}) \end{array}$$ (24)

where *e*_1,r_ = *y*_1,r_ − *x*_1,i_, *e*_1,i_ = *y*_1,i_+*x*_1,r_, *e*_2,r_ = *y*_2,r_ − *x*_2,i_, *e*_2,i_ = *y*_2,i_+*x*_2,r_, *e*_3_ = *y*_3_+*x*_3_, *e*_3_ = *y*_4_ − *x*_3_.

**Fig 7:**
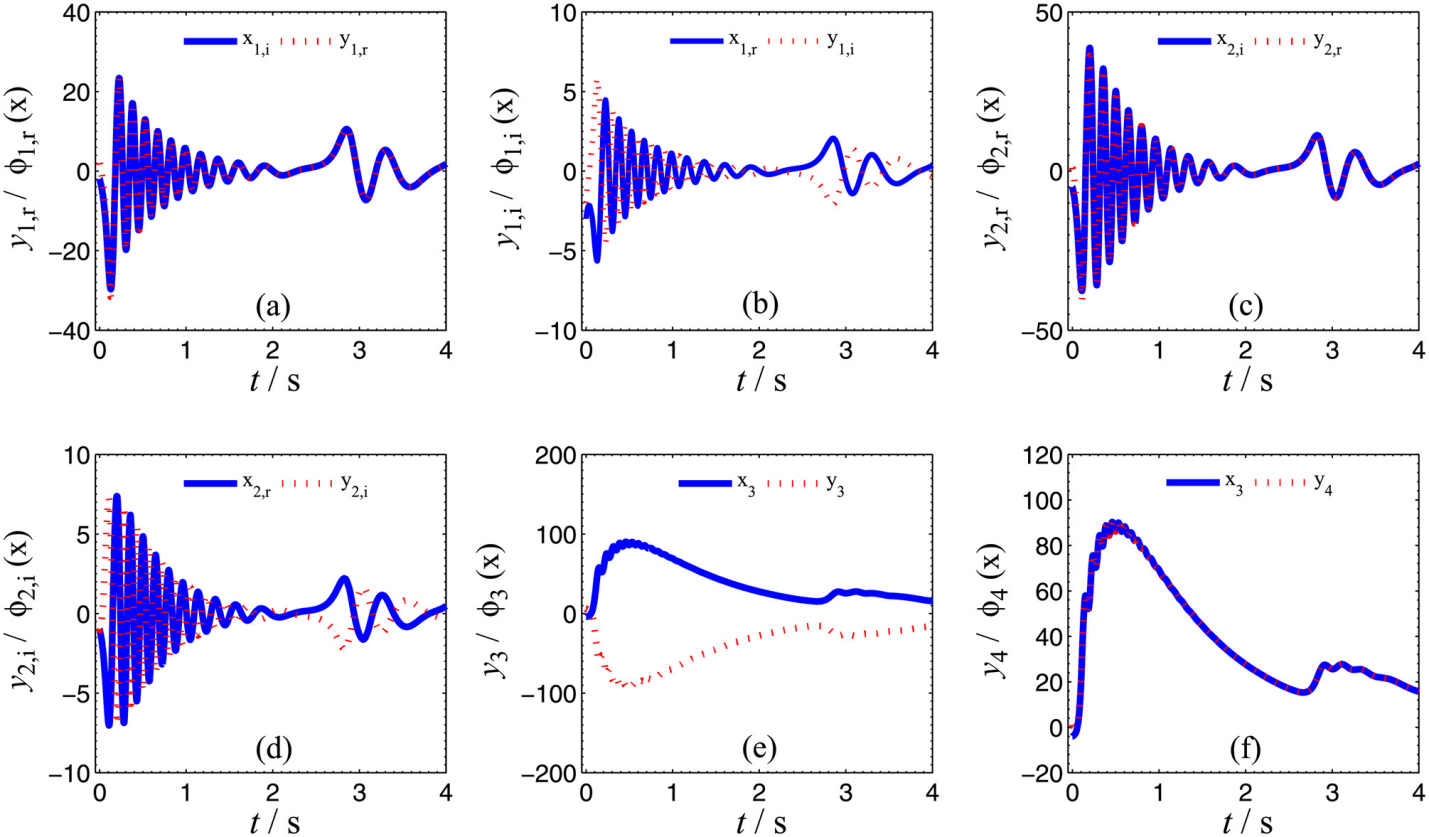
CGS process of systems (19) and (13) with respect to the given complex map vector *ϕ*(*x*) = [−j*x*_1_, −j*x*_2_, −*x*_3_, *x*_3_]^*T*^. (a) *x*_1,i_, *y*_1,r_; (b) *x*_1r_, *y*_1,i_; (c) *x*_2,i_, *y*_2,r_; (d) *x*_2,r_, *y*_2,i_; (e) *x*_3_, *y*_3_; (f) *x*_3_, *y*_4_.

**Fig 8:**
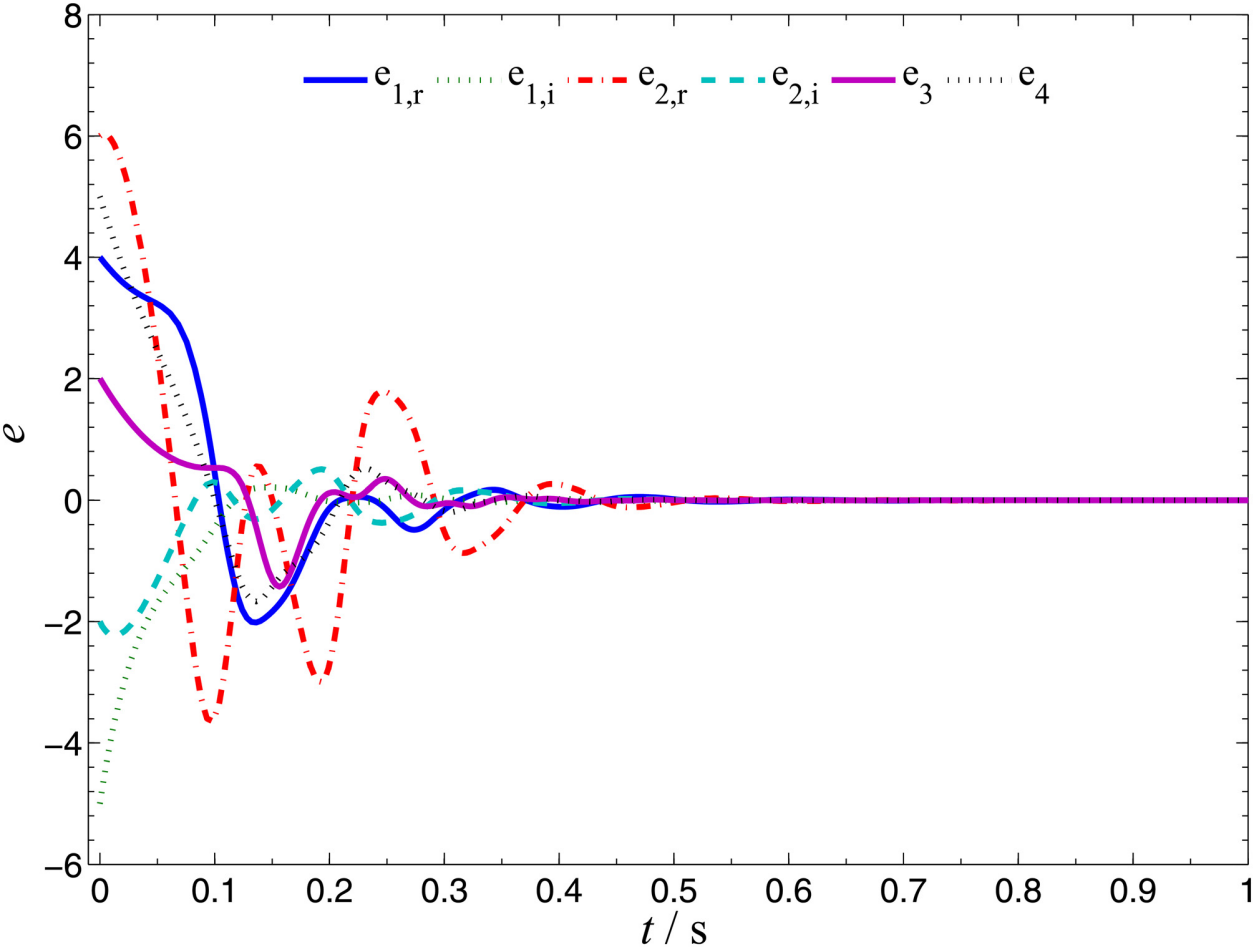
CGS errors of systems (19) and (13).

**Fig 9:**
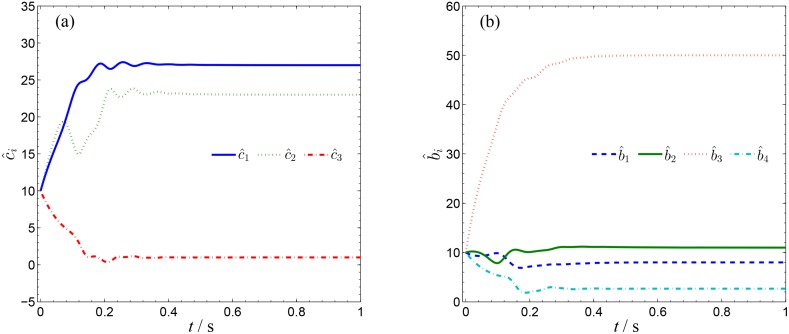
Identification of unknown parameters of systems (19) and (13). (a) ${\hat{c}}_{\text{1}},\;{\hat{c}}_{\text{2}},\;{\hat{c}}_{\text{3}}\;$; (b) ${\hat{b}}_{\text{1}},\;{\hat{b}}_{\text{2}},\;{\hat{b}}_{\text{3}},\;{\hat{b}}_{\text{4}}\;$.

Numerical simulations are presented to verify the validity and effectiveness of CGS between systems (19) and (13) with respect to the complex vector (20), under the following parameter configurations and initial conditions: the known parameters *α*_2_ = 0.67 × 10^−3^, *β*_2_ = 0.02 × 10^−3^, the true values of unknown parameters *θ* = [27,23,1]^*T*^, *δ* = [8, 11, 50, 8/3]^*T*^, the initial conditions of system (19) and (13) ***x***(o) = [−3 − 2j, −1 − 5j, −4]^*T*^, ***y***(0) = [2 − 2j, 1 − j, 6, 1]^*T*^, the initial values of unknown parameters $\hat{\theta }(0)={\left[10,\;10,\;10,\;10\right]}^{T}$, $\hat{\delta }(0)={\left[10,\;10,\;10\right]}^{T}$, and the control strength ***K*** = diag(20, 20, 20, 20), ***K***_*θ*_ = diag(10, 10, 10, 10), ***K***_*δ*_ = diag(10, 10, 10, 10). The corresponding simulation results are shown in Figs 7, 8 and 9. The CGS process is plotted in Fig 7, from which one can see that *y*_1,r_, *y*_2,r_, *y*_4_ are synchronized with *x*_1,i_, *x*_2,i_, *x*_3_, and *y*_1,i_, *y*_2,i_, *y*_3_ are antisynchronized with *x*_1,r_, *x*_2,r_, *x*_3_. The synchronization errors, as shown in Fig 8, converge to zero in a short time. Fig 9 shows the identifying processes of unknown parameters, which indicates that the estimated values tend to be their true values adaptively, i.e., $\hat{\theta }\to {\left[27,\;23,\;1\right]}^{T}$ and $\hat{\delta }\to {\left[8,\;11,\;50,\;2.667\right]}^{T}$.

## CGS of a Memristor-Based Hyperchaotic Complex Lü System and a Chaotic Complex Lü System (n>m)

**Fig 10:**
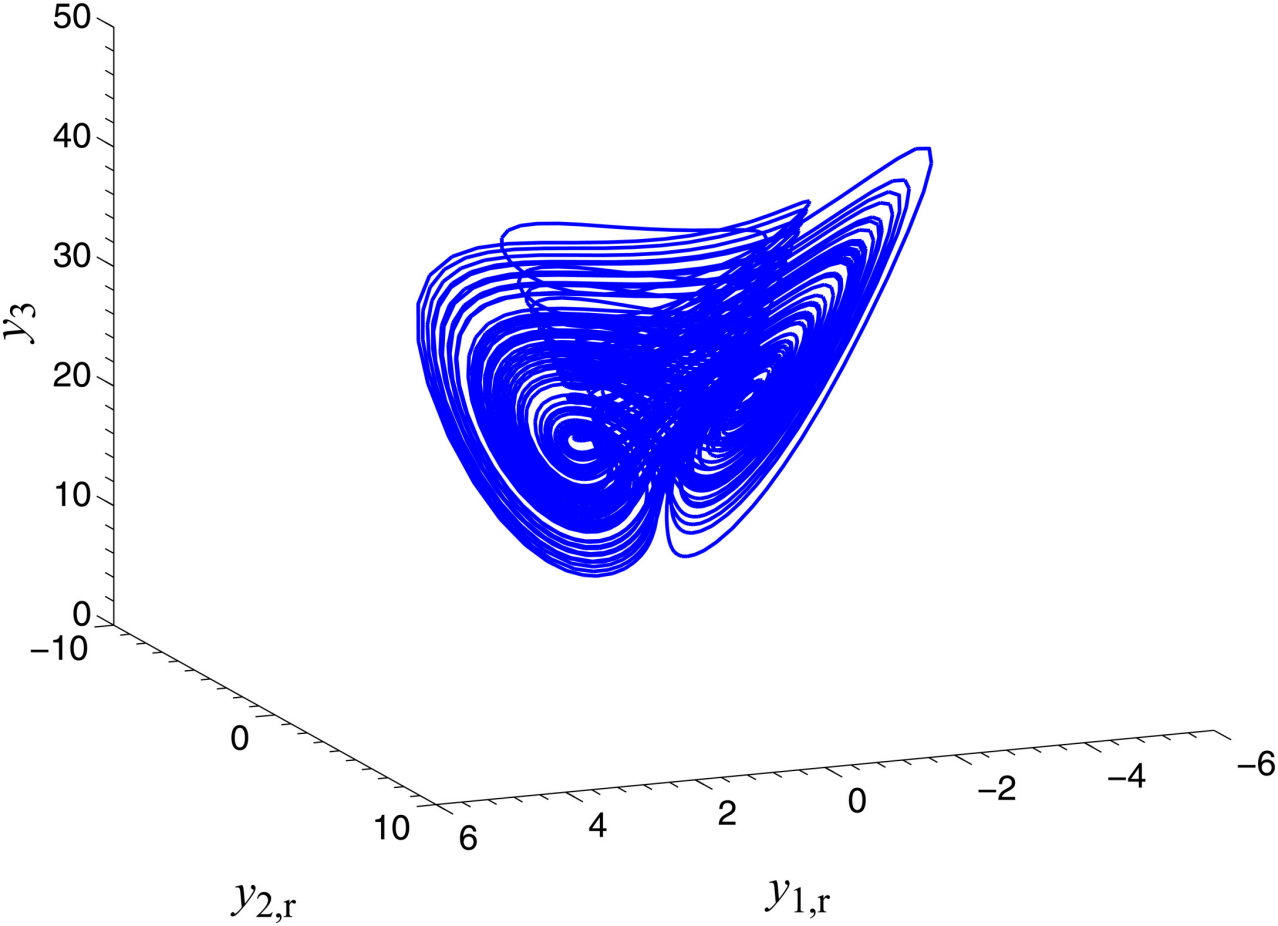
Chaotic attractor of the complex Lü system.

In this section, we investigate CGS of two nonidentical complex systems via reduced order. The memristor-based hyperchaotic complex Lü system, i.e., system (12), is acted as the drive complex system. And a chaotic complex Lü system, investigated in [9], is introduced as the response complex system, which is described as

$$\{\begin{array}{l} {\dot{y}}_{1}=d_{1}(y_{2}-y_{1})+u_{1}\\ {\dot{y}}_{2}=-y_{1}y_{3}+d_{2}y_{2}+u_{2}\\ {\dot{y}}_{3}=\frac{1}{2}({\bar{y}}_{1}y_{2}+y_{1}{\bar{y}}_{2})-d_{3}y_{3}+u_{3} \end{array}$$ (25)

where *y*_1_, *y*_2_∈C, *y*_3_∈R, *d*_1_, *d*_2_ and *d*_3_ are unknown real parameters, *u*_1_, *u*_2_ and *u*_3_ are controllers. When *d*_1_ = 29, *d*_2_ = 21, *d*_3_ = 2, and *y*(0) = [4 + 10j, 6 + 10j, 12]^*T*^, the complex Lü system (25) operates chaotically without control, as shown in Fig 10.

The drive system (12) and response system (25) can be rewritten with the form of Eqs 1 and 2, where ***θ*** = [*a*_1_, *a*_2_, *a*_3_, *a*_4_]^*T*^, ***δ*** = [*d*_1_, *d*_2_, *d*_3_]^*T*^, ***u*** = [*u*_1_, *u*_2_, *u*_3_]^*T*^, and

$$F(x)=\left[\begin{array}{cccc} x_{\text{2}}-x_{\text{1}} & 0 & 0 & 0\\ 0 & x_{\text{2}} & -(\alpha_{1}+3\beta_{1}x_{4}^{2})x_{\text{1}} & 0\\ 0 & 0 & 0 & -x_{3}\\ 0 & 0 & 0 & 0 \end{array}\right],\;f(x)=\left[\begin{array}{c} 0\\ -x_{\text{1}}x_{3}\\ (x_{\text{1}}{\bar{x}}_{\text{2}}+{\bar{x}}_{\text{1}}x_{\text{2}})/2\\ (x_{\text{1}}+{\bar{x}}_{\text{1}})/2 \end{array}\right]$$

$$G(y)=\left[\begin{array}{ccc} y_{2}-y_{1} & 0 & 0\\ 0 & y_{2} & 0\\ 0 & 0 & -y_{3} \end{array}\right],\;g(y)=\left[\begin{array}{c} 0\\ -y_{\text{1}}y_{3}\\ (y_{\text{1}}{\bar{y}}_{\text{2}}+{\bar{y}}_{\text{1}}y_{\text{2}})/2 \end{array}\right]$$

The complex map vector is given by

$$\phi (x)={\left[\text{j}x_{2},\text{j}x_{1},x_{3}-x_{4}^{2}\right]}^{T}$$ (26)

The Jacobian matrix of the complex map vector is calculated as

$$J(\phi )=\left[\begin{array}{llll} 0 & j & 0 & 0\\ j & 0 & 0 & 0\\ 0 & 0 & 1 & -2x_{4} \end{array}\right]$$ (27)

According to Eqs 7 and 8, the complex adaptive controller and parameter estimator can be obtained as

$$\begin{array}{c} u_{1}=[x_{\text{1,i}}x_{3}-x_{\text{2,i}}{\hat{a}}_{2}+(\alpha_{1}+3\beta_{1}x_{4}^{2})x_{\text{1,i}}{\hat{a}}_{3}-(y_{\text{2,r}}-y_{\text{1,r}}){\hat{d}}_{1}-k_{1}e_{\text{1,r}}]\\ \;+\text{j }[-x_{\text{1,r}}x_{3}+x_{\text{2,r}}{\hat{a}}_{2}-(\alpha_{1}+3\beta_{1}x_{4}^{2})x_{\text{1,r}}{\hat{a}}_{3}-(y_{\text{2,i}}-y_{\text{1,i}}){\hat{d}}_{1}-k_{1}e_{\text{1,i}}]\\ u_{2}=[y_{\text{1,r}}y_{3}-(x_{\text{2,i}}-x_{\text{1,i}}){\hat{a}}_{1}-y_{\text{2,r}}{\hat{d}}_{2}-k_{2}e_{\text{2,r}}]\\ \;+\text{j }[y_{\text{1,i}}y_{3}+(x_{\text{2,r}}-x_{\text{1,r}}){\hat{a}}_{1}-y_{\text{2,i}}{\hat{d}}_{2}-k_{2}e_{\text{2,i}}]\\ u_{3}=-y_{\text{1,r}}y_{\text{2,r}}-y_{\text{1,i}}y_{\text{2,i}}+x_{\text{1,r}}x_{\text{2,r}}+x_{\text{1,i}}x_{\text{2,i}}-2x_{\text{1,r}}x_{4}-x_{3}{\hat{a}}_{4}+y_{\text{3}}{\hat{d}}_{3}-k_{3}e_{\text{3}} \end{array}$$ (28)

$$\begin{array}{l} {\dot{\hat{a}}}_{1}=(x_{\text{2,i}}-x_{\text{1,i}})e_{\text{2,r}}-(x_{\text{2,r}}-x_{\text{1,r}})e_{\text{2,i}}-k_{\theta 1}({\hat{a}}_{1}-a_{1})\\ {\dot{\hat{a}}}_{2}=x_{\text{2,i}}e_{\text{1,r}}-x_{\text{2,r}}e_{\text{1,i}}-k_{\theta 2}({\hat{a}}_{2}-a_{2})\\ {\dot{\hat{a}}}_{3}=(-x_{\text{1,i}}e_{\text{1,r}}+x_{\text{1,r}}e_{\text{1,i}})(\alpha_{1}+3\beta_{1}x_{4}^{2})-k_{\theta 3}({\hat{a}}_{3}-a_{3})\\ {\dot{\hat{a}}}_{4}=x_{\text{3}}e_{\text{3}}-k_{\theta 4}({\hat{a}}_{4}-a_{4}) \end{array}$$ (29)

$$\begin{array}{l} {\dot{\hat{d}}}_{1}=(y_{\text{2,r}}-y_{\text{1,r}})e_{\text{1,r}}+(y_{\text{2,i}}-y_{\text{1,i}})e_{\text{1,i}}-k_{\delta 1}({\hat{d}}_{1}-d_{1})\\ {\dot{\hat{d}}}_{2}=y_{\text{2,r}}e_{\text{2,r}}+y_{\text{2,i}}e_{\text{2,i}}-k_{\delta 2}({\hat{d}}_{2}-d_{2})\\ {\dot{\hat{d}}}_{3}=-y_{\text{3}}e_{\text{3}}-k_{\delta 3}({\hat{d}}_{3}-d_{3}) \end{array}$$ (30)

where $e_{\text{1,r}}=y_{\text{1,r}}+x_{\text{2,i}},e_{\text{1,i}}=y_{\text{1,i}}-x_{\text{2,r}},e_{\text{2,r}}=y_{\text{2,r}}+x_{\text{1,i}},e_{\text{2,i}}=y_{\text{2,i}}-x_{\text{1,r}},e_{\text{3}}=y_{\text{3}}-x_{\text{3}}+x_{4}^{2}$.

**Fig 11:**
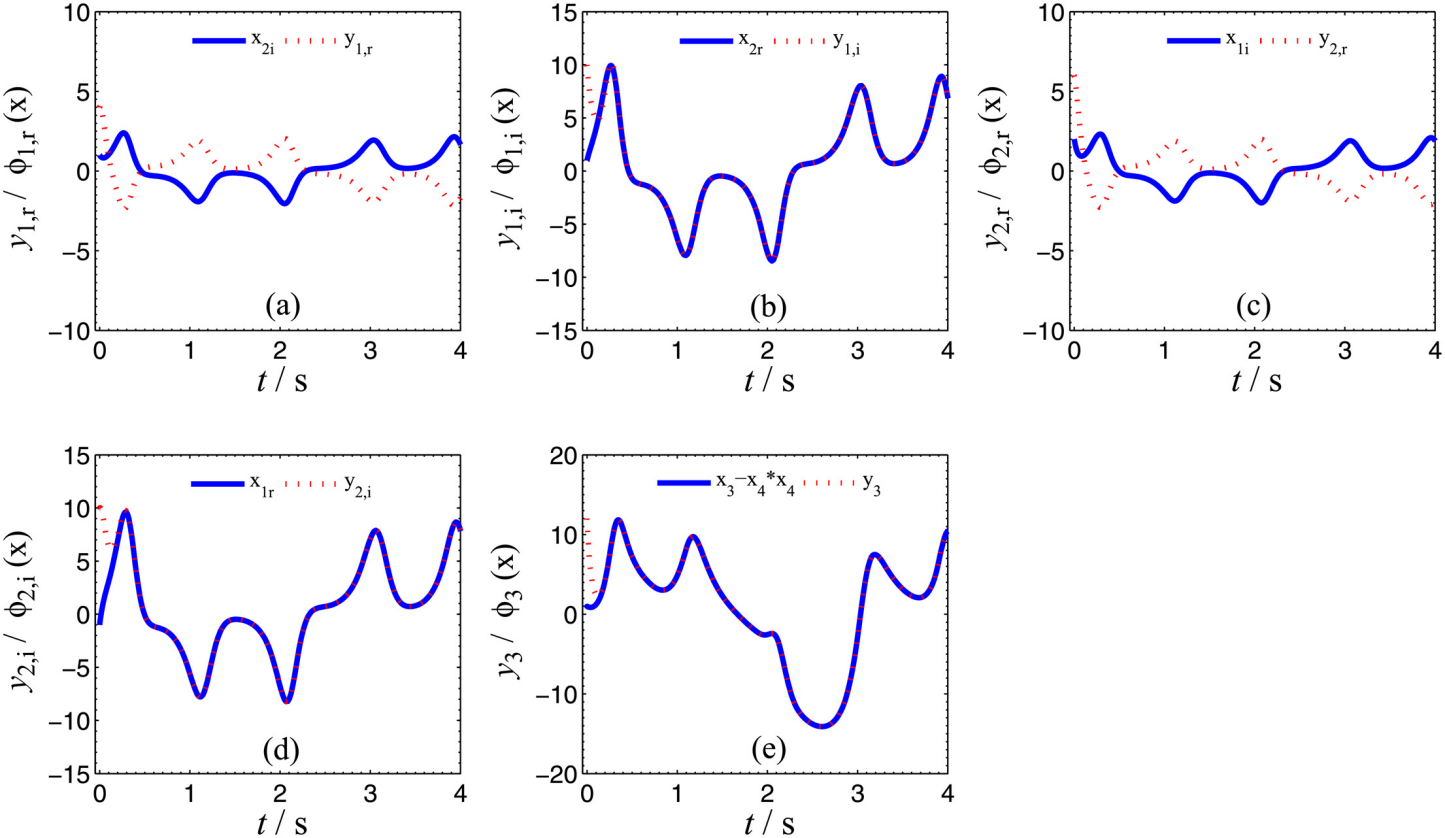
CGS process of systems (12) and (25) with respect to the given complex map vector $\phi (x)={\left[\text{j }x_{2},\text{ j }x_{1},\;x_{3}-x_{4}^{2}\right]}^{T}$. (a) *x*_2,i_, *y*_1,r_; (b) *x*_2r_, *y*_1,i_; (c) *x*_1,i_, *y*_2,r_; (d) *x*_1,r_, *y*_2,i_; (e)$x_{\text{3}}-x_{4}^{2},\;y_{\text{3}}$.

**Fig 12:**
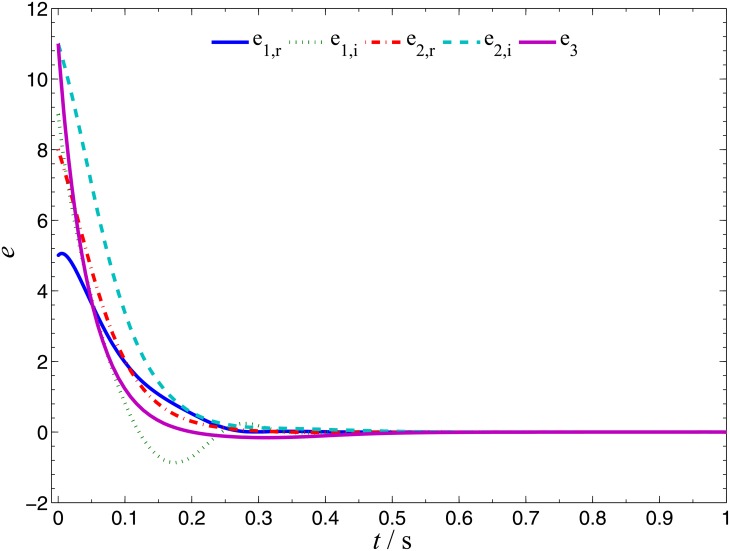
CGS errors of systems (12) and (25).

**Fig 13:**
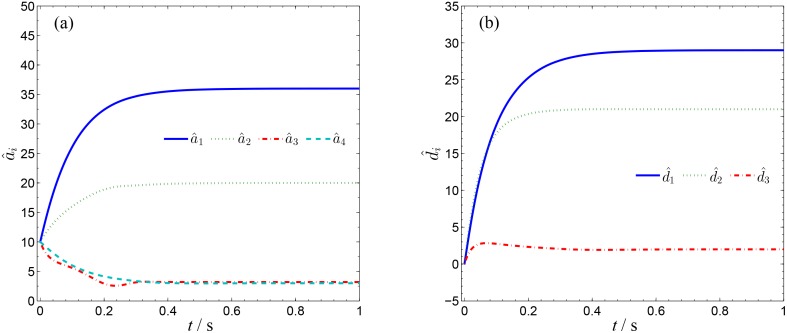
Identification of unknown parameters of systems (12) and (25). (a) ${\hat{a}}_{\text{1}},\;{\hat{a}}_{\text{2}},\;{\hat{a}}_{\text{3}},\;{\hat{a}}_{\text{4}}\;$; (b) ${\hat{d}}_{\text{1}},\;{\hat{d}}_{\text{2}},\;{\hat{d}}_{\text{3}},\;{\hat{d}}_{\text{4}}\;$.

In order to verify the validity and effectiveness of CGS between Systems (12) and (25) with respect to the complex vector (26), simulation results are presented in Figs 11, 12 and 13, with the following parameters and initial conditions: *α*_1_ = 4, *β*_1_ = 0.01, *θ* = [36, 20, 3.2, 3]^*T*^, *δ* = [29,21,2]^*T*^, *x*(0) = [−1 + 2j, 1 + j, 2, −1]^*T*^, ***y***(0) = (4 + 10j, 6 + 10j, 12),$\hat{\theta }(0)={\left[10,\;10,\;10,\;10\right]}^{T}$,$\hat{\delta }(0)={\left[0,\;0,\;0\right]}^{T}$,***K*** = diag(20, 20, 20, 20), ***K***_*θ*_ = diag(10, 10, 10, 10), ***K***_*δ*_ = diag(10, 10, 10, 10). The CGS process is plotted in Fig 11, from which one can see that *y*_1,r_, *y*_2,r_ are antisynchronized with *x*_2,i_, *x*_1,i_, *y*_1,i_, *y*_2,i_ are synchronized with *x*_2,r_, *x*_1,r_, and *y*_3_ is synchronized with $x_{\text{3}}-x_{4}^{2}$, respectively. The synchronization errors converge to zero quickly, as shown in Fig 12. Fig 13 shows that the estimated values of the unknown parameters tend to be their true values adaptively, i.e., $\hat{\theta }\to {\left[36,\;20,\;3.2,\;3\right]}^{T}$ and $\hat{\delta }\to {\left[29,\;21,\;2\right]}^{T}$.

## Conclusions

This paper investigates a novel synchronization scheme named complex generalized synchronization, and its application to synchronization and parameter identification of two nonidentical complex nonlinear systems with fully unknown parameters. An adaptive controller and a parameter estimator are proposed and proved theoretically based on Lyapunov stability theory. Three illustrative examples are presented to verify the correctness and effectiveness of the proposed scheme, namely, CGS of a memristor-based hyperchaotic complex Lü system and a memristor-based chaotic complex Lorenz system, CGS of a chaotic complex Chen system and a memristor-based chaotic complex Lorenz system, as well as CGS of a memristor-based hyperchaotic complex Lü system and a chaotic complex Lü system. The proposed CGS scheme has some advantages, for instance, it can be applied to synchronize complex systems with different orders (generalizability), can be transformed to other types of synchronization with different given complex map vectors (feasibility), can be achieved in a short time with the appropriate control strength (timelines), and can be almost impossibly predicted with the complex map vector (security). So, CGS has extensively potential applications to secure communication, digital cryptography, and so on, which will be involved in our future works.
